# Knowledge, Attitude, and Practices Associated With COVID-19 Among Healthcare Workers in Hospitals: A Cross-Sectional Study in India

**DOI:** 10.3389/fpubh.2021.787845

**Published:** 2021-11-26

**Authors:** Shivkumar Gopalakrishnan, Sangeetha Kandasamy, Bobby Abraham, Monika Senthilkumar, Omar A. Almohammed

**Affiliations:** ^1^Department of Internal Medicine, Government Villupuram Medical College & Hospital, Villupuram, India; ^2^Department of Biochemistry, Government Sivagangai Medical College & Hospital, Sivagangai, India; ^3^Department of Clinical Pharmacy, College of Pharmacy, King Saud University, Riyadh, Saudi Arabia; ^4^Pharmacoeconomics Research Unit, College of Pharmacy, King Saud University, Riyadh, Saudi Arabia

**Keywords:** knowledge, attitude, practice, COVID-19, healthcare workers, India

## Abstract

Severe acute respiratory syndrome coronavirus 2 (SARS-CoV-2) pandemic has caused phenomenal loss of lives and overburdened the health system in India. Low morale, fatigue, and inadequate knowledge among the healthcare workers (HCWs) are the perceived threats to pandemic control. We aimed to assess the COVID-19 related level of knowledge, attitude, and practices (KAP) among our HCWs. A cross-sectional, electronically distributed, questionnaire-based study was conducted which identified the demographics of HCWs and the current KAP related to coronavirus disease 2019 (COVID-19). The descriptive statistics were used to present the demographics of the participants and chi-square test was used to assess the differences in KAP among the participants. Of 1,429 total participants, 71.9% belonged to age group 21–40 years. Only 40.2% received any infection control training and 62.7% relied upon single source of information update. However, 82.9% of the participants had adequate knowledge. Being married, urban dwelling, and higher qualification were associated with knowledge adequacy (*p* < 0.001). Interestingly, the senior HCWs (age 41–50 years) were least likely to have adequate knowledge (74.1%). About 84% had positive attitude toward COVID-19, but 83.8% of the participants feared providing care to the patients with COVID-19. However, 93% of HCWs practiced safety precautions correctly most of the times and training had no influence on practice. In conclusion, more than 80% of HCWs in the study had adequate knowledge, positive attitude, and practiced safely most of the time. However, the pitfalls, such as poor training, knowledge uncertainties, and fear of disease acquisition among the HCWs need to be addressed.

## Introduction

The pandemic caused by Severe Acute Respiratory Syndrome Coronavirus 2 (SARS-CoV-2) has swept through the world causing unprecedented loss of lives and livelihood. Successful outbreak containment requires sound knowledge of the disease and a positive attitude among affected population. Research efforts in China revealed that an optimistic attitude among general population was conducive to gain victory over coronavirus disease 2019 (COVID-19) pandemic (1).

India is the second most populous country on the globe with a population density of 382 persons/square km (2). Recent updates reveal a case load of 33,289,579 with 443,213 COVID-19 deaths; the second highest in the world in the number of cases and the third in the number of deaths (3). Overcrowding and low literacy level establishes India as a fertile ground for COVID-19 propagation. The knowledge, attitude, and practice (KAP) of key stakeholders influence the dynamics of pandemic behavior (4). The human community has seen epidemics before which have evoked anxiety and fear responses among the affected populations (5). Poor knowledge and improper practices of the hospital workers propagate rather than contain the infection. Furthermore, going to war with a tired and demoralized work force spells catastrophe for the healthcare system. As our country battles against the pandemic, KAP of the healthcare workers (HCWs') need to be assessed to identify the knowledge gaps and gauge the psychological impact on them.

The previous research works have either unveiled the KAP of general public or investigated the mindset of the isolated groups (students, doctors, patients, etc.) preferentially (1, 6, 7). The information available so far is piecemeal which cannot be construed into a meaningful representation of the wholesome healthcare team. Therefore, this study was conducted to assess the COVID-19 related KAP for the entire healthcare team.

## Materials and Methods

### Study Method and Participants

A cross-sectional questionnaire-based study was conducted to assess the KAP related to COVID-19 among the HCWs in India. The questionnaire was constructed and distributed simultaneously to multiple COVID-19 treatment centers across the country. All HCWs in the age group of 21–70 years and working in the COVID-19 treatment centers were considered eligible for the study. They were encouraged to answer the questionnaire distributed electronically and those with poor access or ability to use technology were recruited through printed version of the questionnaire. Their responses were recorded strictly on the basis of anonymity to avoid social desirability bias.

### Questionnaire

A questionnaire was specifically designed for the study using the fixed response questions, both multiple choice and yes/no types with two parts. Part 1 probed the demographic particulars of responders and their consent to participate. Only those who gave consent could access the second part of the questionnaire. Part 2 contained questions assessing knowledge (nine items), attitude (eight items), and practices (seven items) of the HCW. The questionnaire was synthesized, scaled, and scrutinized by the study team in cognizance with field experts. The knowledge items covered aspects about the virus, epidemiology, disease pathology, clinical features, and management. The attitude items covered personal perceptions and attitude, such as fear, insecurity, optimism, confidence, and responsibility. The practice items covered appropriate usage of personal protective equipment (PPE) at work and personal/social life. The questionnaire was previously validated and published by part of the research team in Saudi Arabia. Ten researchers from different specialties have individually and collaboratively revised the items on the questionnaire to ensure its face validity for HCWs from different fields and at different levels. In addition, the internal reliability for the KAP scales were acceptable with a Cronbach's α of 0.7, 0.6, and 0.8, respectively. Further details on the scale validity and scoring were presented in the study that was conducted by part of the team in Saudi Arabia (8).

The questionnaire was piloted among the first 50 participants and further refined based on feedback. An electronic survey tool (Google forms) was used to distribute the online survey. The link for accessing the questionnaire was disseminated using e-mail, WhatsApp, and text messages to reach to the HCWs across the nation. Survey tool mandated the participants to respond to all the questions without which the forms could not complete submission process. Furthermore, the questionnaires were designed both in English and the predominant regional language to overcome the language barrier.

### Statistical Analysis

The descriptive statistics were used to present the demographics of participants and frequencies of personnel with adequate knowledge, positive attitude, and applying safety practices most of the time. The distribution of the KAP of participants based on their demographics was compared using chi-square test. The α level < 0.05 was used for statistical significance. The data from the questionnaire were coded and analyzed using the SAS software, version 9.4 (SAS Institute Inc., Cary, NC, USA).

## Results

A total of 1,429 HCWs completed the questionnaire. Most responders belonged to age group 21–40 years (71.9%) and women outnumbered men (61.5 vs. 38.5%). About 80.6% of all the participants were healthcare professionals (HCPs) [physicians, nurses, pharmacists, and laboratory experts], the remaining 19.4% being non-professional HCW [nursing assistants, para-clinical technicians, and sanitary workers] to represent the supportive staff in the centers. The demographics of participants are presented in Table 1. Notably, only 40.2% of the participants had received any infection control training. Additionally, 62.7% of the participants relied solely upon single source of information and the most common source of information was news media channels (22.8%).

**Table 1:** The participants demographical characteristics.

**Variables**	**Number (%)**
**Age**	
21–30 years	576 (40.3)
31–40 years	451 (31.6)
41–50 years	270 (18.9)
51–60 years	102 (7.1)
61–70 years	30 (2.1)
**Gender**	
Male	550 (38.5)
Female	879 (61.5)
**Residential settlement area**	
Urban	778 (54.4)
Rural	651 (45.6)
**Marital status**	
Married	461 (32.3)
Single	968 (67.7)
**Highest educational qualification**	
High school or less	266 (18.6)
Bachelor or associate degree	337 (23.6)
Master degree	372 (26.0)
Professional or doctoral degree	454 (31.8)
**Health care professional (HCP)**	
No	277 (19.4)
Yes	1,152 (80.6)
**Hospital department for health care professionals** **(***n*** = 1,152)**	
Medical	586 (50.9)
Surgical	148 (12.8)
Nursing	203 (17.6)
Laboratory services	138 (12.0)
Other para-clinical services	49 (4.3)
Pharmacy	28 (2.4)

*Data presented as frequency (%)*.

### Knowledge

The participants answering right to knowledge questions ranged between 60.0 and 99.7% (Table 2). The question with the least correct response was “K6: Multiple proven curative treatment options are available now for COVID-19 all over the world” for which only 60.0% responded correctly. Interestingly, 30% of the participants still believed that the pandemic would end in summer because of high temperatures and humidity. Overall, adequate knowledge was documented among 82.9% of the participants. However, only 74.1% of HCW aged 41–50 years had adequate knowledge. Being married, urban dwelling, and higher educational qualification were associated with a higher probability of knowledge adequacy. Paradoxically, receiving an infection control training had a negative impact on the knowledge scores (77.7 vs. 86.4%, *p* < 0.01).

**Table 2:** Frequency and percentage of the participants with correct responses to the knowledge items on the questionnaire.

**Questionnaire items**	**Number (%)**
K1. COVID-19 is a contagious disease that is caused by?	1,425 (99.7)
K2. The most common manifestation for the COVID-19 is?	1,400 (97.9)
K3. The disease can easily spread through?	1,419 (99.3)
K4. What is the longest incubation period for COVID-19 before experiencing any symptoms?	1,319 (92.3)
K5. Severe cases and death are more common among?	1,198 (83.8)
K6. Multiple proven curative treatment options are available now for COVID-19 all over the world?	858 (60.0)
K7. Most COVID-19 cases are mild and can recover with no treatment?	1,201 (84.0)
K8. We know that the pandemic will be over by summer, as the causative microbe is sensitive to high temperature and humidity?	994 (69.6)
K9. Washing hands with soap and water is effective in eliminating the causative microbe.	1,359 (95.1)

*Data presented as frequency (%)*.

### Attitude and Practices

The participants with positive responses to the attitude questions ranged between 16.2 and 96.6% (Table 3). The item “A3: I think people who got infected with COVID-19, including health care personnel, were infected due to negligence,” evoked a strong agreement from 52.6% of responders. Moreover, 83.8% of the participants feared approaching a patient with COVID-19 despite using PPE. In practice items, the participants following safety practices most of the time ranged between 77.5 and 96.1% (Table 4). The lowest response rate was for Part 2, regarding the right sequence of donning PPE, as only 77.5% of participants were doing it correctly.

**Table 3:** Frequency and percentage of the participants with positive responses to the attitude items on the questionnaire.

**Questionnaire items**	**Number (%)**
A1. In my opinion, all people in the healthcare system and the community are part of this battle against COVID-19, and should be responsible about their role.	1,381 (96.6)
A2. I believe that early detection of COVID-19 cases through mass testing will facilitate or accelerate the control of the COVID-19 pandemic.	1,271 (88.9)
A3. I think people who got infected with COVID-19, including health care personnel, were infected due to negligence *(Reversely scored)*.	677 (47.4)
A4. You have a feel of threat or fear when you become close or provide care to a confirmed or suspected COVID-19 patient *(Reversely scored)*.	232 (16.2)
A5. I think COVID-19 is just a communicable disease which is being given undue importance *(Reversely scored)*.	1,003 (70.2)
A6. I think restricting travels, locking cities, and quarantining all suspected cases are an exaggeration for the current situation *(Reversely scored)*.	1,010 (70.7)
A7. The country's efforts will succeed in the battle against COVID-19 pandemic.	1,048 (73.3)
A8. I think when COVID-19 pandemic is over many benefits and good things will be seen.	1,013 (70.9)

*Data presented as frequency (%)*.

**Table 4:** Frequency and percentage of the participants who were practicing appropriately based on responses to the questionnaire items.

**Questionnaire items**	**Number (%)**
P1. If I or anyone close to me develop any COVID-19 symptoms, I will seek or recommend to others to seek medical attention.	1,347 (94.3)
P2. When I am putting on the personal protective equipment (PPE), I follow the following order: Suit—Mask—Goggles—Gloves.	1,108 (77.5)
P3. I have been careful not to carry my mobile phone/pen, etc.… inside the COVID-19 ward.	1,291 (90.1)
P4. I do not go out unless it is necessary.	1,373 (96.1)
P5. When I finish my shift, I dispose the PPE and scrub thoroughly before entering home/quarters.	1,365 (95.5)
P6. I sanitize my hands with alcohol-based solution before attending to each patient.	1,333 (93.3)
P7. After using my PPE, I dispose them in the appropriate color-coded bins.	1,365 (95.5)

*Data presented as frequency (%)*.

Most of the participants demonstrated a positive attitude (84.2%) toward COVID-19, and practiced safety precautions appropriately (93.0%). Senior HCW aged 51–60 years had more appreciable attitude scores (*p* = 0.009), and young ones (21–30 years) were the least to follow the safety practices (*p* < 0.001). Hitherto female sex, higher qualification, and affiliation to the clinical departments exerted a significant influence toward positive attitude (*p* < 0.001). The married participants were least likely to have positive attitude or follow the safety practices adequately (*p* < 0.001). The demographic distribution of KAP among the study responders are depicted in Table 5.

**Table 5:** Distribution of adequate knowledge, positive attitude, and appropriate practices based on the demographics and characteristics of the participants.

**Variables**	**Category**	**Adequate knowledge (7/9 points)**		**Positive attitude (9/16 points)**		**Appropriate practices (11/14 points)**	
		***N*** **(%)**	p^*^	***N*** **(%)**	***p***	***N*** **(%)**	***p***
Overall		1,185 (82.9)	–	1,203(84.2)		1,329 (93.0)	
Age (in years)	21–30	491 (85.2)	**<0.001**	475 (82.5)	**0.009**	509 (88.4)	**<0.001**
	31–40	386 (85.6)		393 (87.1)		428 (94.9)	
	41–50	200 (74.1)		221 (81.9)		262 (97.0)	
	51–60	83 (81.4)		93 (91.2)		102 (100)	
	61–70	25 (83.3)		21 (70.0)		28 (93.33)	
Gender	Male	455 (82.8)	0.875	418 (76.0)	**<0.001**	512 (93.1)	0.917
	Female	730 (83.1)		785 (89.3)		817 (93.0)	
Marital status	Single	777 (80.3)	**<0.001**	835 (86.3)	**0.002**	932 (96.3)	**<0.001**
	Married	408 (88.5)		368 (79.8)		397 (86.1)	
Residential area	Urban	703 (90.4)	**<0.001**	665 (85.5)	0.143	723 (92.9)	0.908
	Rural	482 (74.0)		538 (82.6)		606 (93.1)	
Received infection control training	No	783 (86.4)	**<0.001**	712 (83.4)	0.305	786 (92.0)	0.082
	Yes	447 (77.7)		491 (85.4)		543 (94.4)	
Health care professional	No	167 (60.3)	**<0.001**	243 (87.7)	0.059	263 (94.9)	0.158
	Yes	1,018 (88.4)		960 (83.3)		1,066 (92.5)	
Educational achievement	≤High school	152 (57.1)	**<0.001**	228 (85.7)	**<0.001**	258 (97.0)	**<0.001**
	Bachelor or associate	265 (78.6)		250 (74.2)		319 (94.7)	
	Master	338 (90.9)		308 (82.8)		324 (87.1)	
	Doctoral	430 (94.7)		417 (91.9)		428 (94.3)	
Hospital department for HCP (*n* = 1,152)	Medical	533 (91.0)	**<0.001**	500 (85.3)	**<0.001**	531 (90.6)	0.097
	Surgical	137 (92.6)		132 (89.2)		139 (93.9)	
	Nursing	173 (85.2)		162 (79.8)		195 (96.1)	
	Laboratory	124 (89.9)		113 (81.9)		127 (92.0)	
	Other para-clinical	31 (63.3)		31 (63.3)		48 (98.0)	
	Pharmacy	20 (71.4)		22 (78.6)		26 (93.0)	

*Data presented as frequency (%)*.

* *p-values were from chi-squared test and the values in bold represent significant results*.

### KAP for the HCPs vs. Non-professional HCWs

The professionals had better level of knowledge (*p* < 0.001) as compared with the non-professional participants (Table 5). However, they did not differ based on positive attitude and appropriate practices. Interestingly, the HCPs in the clinical departments had significantly better knowledge and positive attitude than those of para-clinical departments (*p* < 0.001). However, there was no significant difference in safety practices between the workers in the clinical and para-clinical department.

## Discussion

The invasion of human race by SARS-CoV-2 has claimed more than 4,636,153 lives (3), besides causing economic devastation of the developing countries. Presently, India is cruising through a very strong second wave of pandemic (3). Realistic appreciation of the situation warrants critical assessment of the mental preparedness of the frontline HCWs who take the heat of the onslaught. We therefore aimed to assess the KAP of Indian HCWs of the COVID-19 treatment centers. We found that 82.9% of the HCWs had adequate knowledge, 84.2% displayed positive attitude, and 93.0% adhered to safety practices most of the times.

In this study a cumulative 82.9% of the participants possessed adequate knowledge about COVID-19, and the HCWs who were married, professionals, urban dwellers, and with higher educational qualification had more probability of adequate knowledge than the rest. Interestingly, it was observed that the HCWs of age group 41–50 years were lagging far behind others in knowledge aspect. The HCWs of this age are generally senior doctors, nursing superintendents, and senior supervisors of the paramedical departments. Probable reason for this deficit could be predominant administrative engagements rather than clinical exposure in this group. Relative knowledge inadequacy among the senior HCWs is a cause for concern as they are decision makers in most institutes. A survey conducted at Mumbai among HCP also revealed only 71.2% adequacy of knowledge and akin to our study, the administrative and paramedical staff performed poorly in knowledge (9). In Pakistan, Saqlain et al. reported that 93% of the HCWs possessed adequate knowledge (10). The knowledge figures appeared less in our survey probably because of confounding effect of 19.4% of the non- professional HCWs.

Our survey showed that 62.7% of the HCWs relied only upon single source of information for knowledge update. Disturbingly, the most frequent source was news media channels (22.8%). In the era of evidence-based medicine, the reliance of HCW on non-scientific sources of information is deemed less beneficial for the patient community. This observation could not be dispensed as a regional phenomenon because Saqlain et al. also endorsed the same finding in his survey (10). Of concern was that only 40.2% of HCWs ever had any infection control training at the time of the study. A similar report from a multicenter study in India highlighted that only 56.18% of doctors received training related to COVID-19 and <50% were satisfied with the quality of the training (11). Ironically training had a significant negative impact on the knowledge scores in our study population (*p* < 0.001). Resource and time shortage make the HCW's training, the Achille's heel of health regulatory bodies, more so during pandemic times. Yet this lacuna cannot be left unaddressed, and we recommend decentralization of training programs at the institute level with feedback evaluation by the Ministry of Health.

Among the significant knowledge gaps, notable ones were regarding the treatment aspects and pandemic myths (K6 and K8 of Table 2). Forty percent of the participants believed that many curative treatment options are available for COVID-19 which reflects poor reliance on authenticated information source. Also, 30.4% believed the pandemic would end in summer due to humid climate. Despite the fact that the WHO houses a section on “Myth Busters” in its official website (3), such misbeliefs floating among the HCWs is worrisome.

This survey identified positive attitude among 84.2% of the responders. Among all, 96.6% confirmed having a sense of responsibility towards their role in the pandemic. It was encouraging to note that 73.3% recorded an optimistic outlook of COVID-19 outcome in our country. However, it was offset by 84.8% of the participants harboring deep rooted fear when caring for the patients with COVID-19. The medical fraternity often find it difficult to come to terms with looming uncertainties during an outbreak. A nationwide survey conducted among the doctors identified depression and anxiety among 35% of the responders (12). The Middle East Respiratory Syndrome (MERS) epidemic of 2012 had seen a phenomenal impact on the psychosocial well-being of the doctors involved in patient care activities (13). Disease acquisition fear among the HCWs jeopardizes delivery of optimal care to the patients and forecasts adverse outcome (14, 15). Singh et al. observed that shortage of resources and endless hours of COVID-19 duties have wreaked havoc on the morale of HCWs in India (11). In our study, we were able to identify the factors conducive to negative attitude which included age (61–70 years), male sex, being married, lower educational qualification, and working in the para-clinical departments (*p* < 0.05). Caregiver fatigue is an ominous sign and we urge the Ministry of Health to look into this need as a priority. Stress relieving maneuvers for the HCWs, such as Yoga, peer group activities, and adequate off-duty hours could be our insurance in this regard.

The study found that 93% of the participants reported good adherence to the safety practices and the relative lack of training did not seem to adversely affect the same. However, the younger aged (21–30 years) and married HCWs were less likely to follow sound safety practice; probably due to inexperience and heightened confidence (*p* < 0.001). The weakest link in the chain was donning procedure which only 77.5% of the HCWs practiced properly. A related study among the doctors in India revealed that 94% used face masks appropriately and 95% regularly resorted to hand hygiene (12). The efforts of the Ministry of Health in promulgating awareness among the HCWs about the safety practices deserves commendation at this juncture. Interestingly, we observed that good practices were uniformly followed by the entire spectrum of HCW population with insignificant differences between the professional and non-professional groups. This was in contradiction to the results observed in Pakistan where the pharmacists adhered to infection control practices more than other HCWs (10). Interestingly, the HCWs with the lowest qualification were the best safety practitioners (*p* < 0.001). This paradox is a pseudo phenomenon since most of these staff are of lower cadre [stretcher bearers, nursing assistants, etc.] who are constantly monitored and perform healthcare activities under supervision.

Majority of the study participants (80.6%) were HCP and only 19.4% constituted lower cadre staff (technicians, nursing assistants, sanitary workers, etc.). Hence, caution should be exercised in extrapolating the findings of this study to the entire HCW population. However, the only difference between the two groups was in the knowledge aspect which is something that we would expect. Furthermore, the study recruited participants working in high case load COVID-19 centers spanning the country. Their KAPs are shaped by intense exposure which distinguishes them from the workers in the non-COVID-19 hospitals and primary care setup. The study results largely reflect the KAP of HCWs involved in the care of patients with COVID-19 and the recommendations therein are applicable to this subset only. Therefore, generalization of these findings to all HCWs would lose validity.

The study found the vast majority of HCWs had adequate level of knowledge, positive attitude, and were practicing safely most of the time. However, there were many uncertainties in the KAP of our target population which need to be resolved. First, infection control training status of the HCWs was far from satisfactory. Second, fear of disease acquisition among the HCWs was recognized and may negatively impact patient care. Finally, considerable demographic heterogeneity was revealed in the KAP of HCWs which can be targetted by focused educational and psychological training programs. Summing up, the study identified important knowledge gaps, attitudinal differences, and practice variations among the HCWs in India which leaves a lot of space for improvement. The authors conclude that there is scope for improvement in this aspect of pandemic control strategy of the country which could pave the way for better outcomes. Remedial measures suggested include targeted training and mental health programs for the HCWs, capacity building of health facilities and curb on social media disseminating fear. With SARS-CoV-2 still on the rampage, it is time that the country takes a second look at the weak links disabling the COVID-19 warriors.

## Data Availability Statement

The raw data supporting the conclusions of this article will be made available by the authors, without undue reservation.

## Ethics Statement

The studies involving human participants were reviewed and approved by Institutional Ethics Committee of Government Villupuram Medical College and Hospital, Tamil Nadu, India. The Ethics Committee waived the requirement of written informed consent for participation.

## Author Contributions

SG, SK, and OA: conceptualization, data curation, and visualization. SG and OA: methodology. OA: software, formal analysis, and funding acquisition. SG, SK, BA, and OA: validation. SG, BA, and MS: investigation and writing—original draft preparation. SK and OA: writing—review and editing. SG: supervision and project administration. All authors have read and agreed to the published version of the manuscript.

## Funding

This research was funded by the Researcher Supporting Project (RSP-2020/77), King Saud University, Riyadh, Saudi Arabia.

## Conflict of Interest

The authors declare that the research was conducted in the absence of any commercial or financial relationships that could be construed as a potential conflict of interest.

## Publisher's Note

All claims expressed in this article are solely those of the authors and do not necessarily represent those of their affiliated organizations, or those of the publisher, the editors and the reviewers. Any product that may be evaluated in this article, or claim that may be made by its manufacturer, is not guaranteed or endorsed by the publisher.

## References

[1] Zhong B-L Luo W Li H-M Zhang Q-Q Liu X-G Li W-T. Knowledge, attitudes, and practices towards COVID-19 among Chinese residents during the rapid rise period of the COVID-19 outbreak: a quick online cross-sectional survey. Int J Biol Sci. (2020) 16:1745–52. 10.7150/ijbs.45221 32226294 PMC7098034

[2] National Institution for Transforming India. State Statistics: Population Density. Available online at: https://www.niti.gov.in/niti/content/population-density-sq-km (accessed August 26, 2020).

[3] World Health Organization. WHO Coronavirus (COVID-19) Dashboard. Available online at: https://covid19.who.int/ (accessed September 15, 2021).

[4] Ajilore K Atakiti I Onyenankeya K. College students' knowledge, attitudes and adherence to public service announcements on Ebola in Nigeria: suggestions for improving future Ebola prevention education programmes. Health Educ J. (2017) 76:648–60. 10.1177/0017896917710969

[5] Person B Sy F Holton K Govert B Liang A. Fear and stigma: the epidemic within the SARS outbreak. Emerg Infect Dis. (2004) 10:358–63. 10.3201/eid1002.030750 15030713 PMC3322940

[6] Olum R Kajjimu J Kanyike AM Chekwech G Wekha G Nassozi DR. Perspective of medical students on the COVID-19 pandemic: survey of nine medical schools in Uganda. JMIR Public Health Surveill. (2020) 6:e19847. 10.2196/19847 32530815 PMC7307324

[7] Tachfouti N Slama K Berraho M Nejjari C. The impact of knowledge and attitudes on adherence to tuberculosis treatment: a case-control study in a Moroccan region. Pan Afr Med J. (2012) 12:52. 10.11604/pamj.2012.12.52.1374 22937192 PMC3428172

[8] Almohammed OA Aldwihi LA Alragas AM Almoteer AI Gopalakrishnan S Alqahtani NM. Knowledge, attitude, and practices associated with COVID-19 among healthcare workers in hospitals: a cross-sectional study in Saudi Arabia. Front Public Health. (2021) 9:643053. 10.3389/fpubh.2021.643053 34368039 PMC8342857

[9] Modi PD Nair G Uppe A Modi J Tuppekar B Gharpure AS. COVID-19 awareness among healthcare students and professionals in mumbai metropolitan region: a questionnaire-based survey. Cureus. (2020) 12:e7514. 10.7759/cureus.7514 32377462 PMC7198075

[10] Saqlain M Munir MM Rehman SU Gulzar A Naz S Ahmed Z. Knowledge, attitude, practice and perceived barriers among healthcare workers regarding COVID-19: a cross-sectional survey from Pakistan. J Hosp Infect. (2020) 105:419–23. 10.1016/j.jhin.2020.05.007 32437822 PMC7211584

[11] Singh H Sharma S. Concerns of frontline doctors in india during COVID-19: a cross-sectional survey. Indian J Public Health. (2020) 64:237–9. 10.4103/ijph.IJPH_472_20 32496264

[12] Chatterjee S Bhattacharyya R Bhattacharyya S Gupta S Das S Banerjee B. Attitude, practice, behavior, and mental health impact of COVID-19 on doctors. Indian J Psychiatry. (2020) 62:257–65. 10.4103/psychiatry.IndianJPsychiatry_333_20 32773868 PMC7368446

[13] Um DH Kim JS Lee HW Lee SH. psychological effects on medical doctors from the middle east respiratory syndrome (MERS) outbreak : a comparison of whether they worked at the MERS occurred hospital or not, and whether they participated in MERS diagnosis and treatment. J Korean Neuropsychiatr Assoc. (2017) 56:28–34. 10.4306/jknpa.2017.56.1.28

[14] Lai J Ma S Wang Y Cai Z Hu J Wei N. Factors associated with mental health outcomes among health care workers exposed to coronavirus disease 2019. JAMA Netw Open. (2020) 3:e203976. 10.1001/jamanetworkopen.2020.3976 32202646 PMC7090843

[15] Tan BYQ Chew NWS Lee GKH Jing M Goh Y Yeo LLL. psychological impact of the COVID-19 pandemic on health care workers in Singapore. Ann Intern Med. (2020) 173:317–20. 10.7326/M20-1083 32251513 PMC7143149

