# Corrigendum: Knowledge, Attitude, and Practices Associated With COVID-19 Among Healthcare Workers in Hospitals: A Cross-Sectional Study in India

**DOI:** 10.3389/fpubh.2022.863368

**Published:** 2022-02-25

**Authors:** Shivkumar Gopalakrishnan, Sangeetha Kandasamy, Bobby Abraham, Monika Senthilkumar, Omar A. Almohammed

**Affiliations:** ^1^Department of Internal Medicine, Government Villupuram Medical College & Hospital, Villupuram, India; ^2^Department of Biochemistry, Government Sivagangai Medical College & Hospital, Sivagangai, India; ^3^Department of Clinical Pharmacy, College of Pharmacy, King Saud University, Riyadh, Saudi Arabia; ^4^Pharmacoeconomics Research Unit, College of Pharmacy, King Saud University, Riyadh, Saudi Arabia

**Keywords:** knowledge, attitude, practice, COVID-19, healthcare workers, India

There is an error in the Funding statement. The correct Name for the Funder is **Researcher Supporting Project (RSP-2020/77), King Saud University, Riyadh, Saudi Arabia**. The name was correct in the FUNDING section but incorrect in the ACKNOWLEDGMENTS section as an additional statement was mistakenly added to the ACKNOWLEDGMENTS section.

The authors apologize for this error and state that this does not change the scientific conclusions of the article in any way. The original article has been updated.

## Publisher's Note

All claims expressed in this article are solely those of the authors and do not necessarily represent those of their affiliated organizations, or those of the publisher, the editors and the reviewers. Any product that may be evaluated in this article, or claim that may be made by its manufacturer, is not guaranteed or endorsed by the publisher.

